# Exploring Client Perceptions on Gaining Infant Feeding Information Through the Texas Women, Infants, and Children (WIC) Chatbot

**DOI:** 10.3390/ijerph22020193

**Published:** 2025-01-29

**Authors:** Kelci Baez, Lesli Biediger-Friedman, Cassandra M. Johnson, Emily Stubblefield, Lizzeth Escalera, Brittany Reese Markides

**Affiliations:** 1 Nutrition and Foods Program, School of Family and Consumer Sciences, Texas State University, San Marcos, TX 78666, USA; kdb181@txstate.edu (K.B.); cassandra_johnson@txstate.edu (C.M.J.); ujf9@txstate.edu (E.S.); l_e92@txstate.edu (L.E.); 2 Institute for Physical Activity and Nutrition, School of Exercise and Nutrition Sciences, Deakin University, Geelong, VIC 3216, Australia; brittany.m@research.deakin.edu.au

**Keywords:** m-health, intervention, nutrition assistance program, health technology, pandemic, public health

## Abstract

The modernization of the Special Supplemental Nutrition Program for Women, Infants, and Children (WIC) program is a priority. The Texas WIC chatbot, Maya, streamlines client interactions through dialog-based responses. This qualitative study explored client capabilities, motivations, and opportunities for seeking nutrition information about breastfeeding, formula feeding, infant feeding safety, adequacy of infant feeding, and complementary feeding via a chatbot. A team conducted in-depth semi-structured interviews with Texas WIC clients (n = 19 women). All interviews were transcribed and subjected to a two-coder, four-phase process utilizing a theory-based codebook. Codes were compiled and thematically categorized. Identified themes included (1) motivations through necessity or resource availability, (2) client capabilities and Maya usability, and (3) opportunities for connection, support, and encouragement. Texas WIC clients that participated in this study expressed motivations, capabilities, and opportunities to engage with nutrition information through Maya. They described Maya as a favorable resource for behavior changes, and a trusted source of nutrition information, citing the credibility of WIC and reliability of the chatbot. The findings may inform future research and development of public health chatbots. Additional research is required to explore how different factors such as language and technology usage may impact client capabilities, motivations, and opportunities to seek nutrition information with regard to infant feeding.

## 1. Introduction

### 1.1. Program Overview

The Special Supplemental Nutrition Program for Women, Infants, and Children (WIC) is a federal grant program administered by the United States Department of Agriculture (USDA). WIC provides eligible program participants, also known as clients, with nutrition-related benefits and services, including food packages, nutrition education, health care referrals, and lactation support [1,2], and includes mandatory nutrition education [3]. Before the COVID-19 pandemic, Texas WIC delivered nutrition education in-person, online, or through phone calls. However, the use of online nutrition education increased during the pandemic and has remained at the same level post-pandemic due to the benefits of having easily accessible information and services [4,5]. Data from the pandemic also highlighted a need to leverage technology to innovate nutrition education, such as by providing clients with online access to trusted nutrition information for infant feeding [4,6]. Recently, Texas WIC has delivered nutrition education primarily via online classes through TexasWIC.org [5,7,8]. In a survey of Texas WIC clients (n = 10,221), respondents preferred to receive future nutrition education online (70.8%) compared to in the WIC clinic (36.5%), over the phone (25.2%), or in the community (6.3%) [7]. These data point to a need for modernization as programs consider evolving in a way that would involve offering more online services.

### 1.2. Need for Modernization

WIC coverage rates have steadily declined for nearly a decade [9]. Current research suggests that enhancing scheduling options, providing appointment reminders, and increasing program information availability and accessibility may improve participation [6,10,11], especially through the investment in technological infrastructure [4,6,12]. Technological solutions are supported by data comparing pre-pandemic and post-pandemic WIC coverage rates [4,11,12]. On average, states that offered technologically remote solutions for gaining and renewing program benefits experienced a participation increase (3.9%), while most states without remote solutions experienced a decrease (7.6%) [11]. The USDA also supports this need for modernization through funding opportunities for technological innovations, which encourage outreach, participation, effectiveness, and satisfaction [6,12,13]. In fact, the 2023 WIC Technology Landscape Report identified existing WIC innovations such as online portals, mobile applications [12,14,15], online videoconferencing [12,16], and chatbots [12,17], and the findings offer support for m-health interventions.

### 1.3. M-Health Opportunities

As the call to modernize WIC is extended, chatbots have emerged as a promising m-health strategy. Chatbots have gained popularity in many settings as a platform for business and communications, connecting people with services, information, and acquaintances [18]. Additionally, Brandtzaeg et al. found that multiple factors motivated people to use chatbots, with productivity being the primary motivator and indicated a need to understand differences in technology users’ motivations [19]. Chatbot technology can alleviate the administrative burden by using human-like conversations to facilitate common tasks, such as registering for a benefit or service, scheduling an appointment, or receiving general information [20,21]. Chatbots can be dialog-based or facilitated via artificial intelligence (AI) programs like ChatGPT. A dialog-based chatbot generates responses based on a preprogrammed set of rules and heuristics devised by human developers and can facilitate ‘question-and-answer’-style conversations [21,22]. Chatbots can be found in a variety of settings, including health care [23,24,25,26,27], lifestyle, wellness [24,28,29,30,31], and public health [32,33]. Previous studies have tested chatbots as digital health, e-health, or m-health interventions [28,29,30,31,32,34,35], but no one had developed or tested a chatbot for maternal or infant nutrition prior to the creation of Maya, the Texas WIC chatbot.

In 2017, Texas WIC led the development, implementation, and evaluation of a dialog-based WIC chatbot named Maya [17,36]. Importantly, this chatbot was the first of its kind in the field of public health nutrition. Maya, the chatbot, helps clients to search for local WIC offices and approved stores, report a lost or stolen card, upload documentation, and answer client questions [17,36]. Currently, the Texas WIC chatbot is a champion for additional WIC chatbots and technological innovations related to public health. Previous chatbot evaluations and unpublished Texas WIC research have indicated that visitors would like to ask Maya nutrition-related questions and use Maya to access nutrition information [17,36]. Specifically, clients identified the following five topics as priorities: breastfeeding, formula feeding, infant feeding safety, adequacy of infant feeding, and complementary feeding [17,36]. Providing content and options for delivery of infant feeding information via chatbot requires development. Previous studies have focused on WIC technological innovations [12,14,15,16,17,37,38,39,40], but because of heterogeneity across the studies and chatbot platforms, the effectiveness of chatbots is not well understood [29,33,41]. The literature offers limited insights into how best to provide nutrition information about infant feeding to clients via a chatbot. Technology can play a critical role in modernizing WIC. Advances continue to demonstrate feasible technological solutions to connect people to information, programs, and services [14,15,16,39]. Public health programs, like WIC, can benefit from technological innovations, efficiently connecting program participants with the utilization of program services [17,36]. Therefore, there is a need for a deeper understanding of WIC clients’ beliefs around regarding a chatbot providing information about infant nutrition. The objective of this qualitative study was to explore the motivations, capabilities, and opportunities of clients seeking nutrition information about infant feeding through Maya, the Texas WIC chatbot. The findings will help inform the content and delivery of nutrition information, which partially addresses the modernization challenges of access and usage of programmatic services.

## 2. Materials and Methods

### 2.1. Study Overview

This study applied a qualitative approach to collect cross-sectional data with remote or online interviews among current Texas WIC participants. Previously, members of this team had developed, implemented, and evaluated the Texas WIC Chatbot, Maya [17,36], based on user-centered design (UCD) [42]. Briefly, UCD is an iterative process that positions technology users and key informants in the following technology developmental phases: (1) ideation and development, (2) deployment, and (3) evaluation [42]. A multi-step process was used to advance the project through multiple rounds of UCD phases. This study engages with phases 1 and 3 of the UCD iterative process.

The Institutional Review Board (IRB) approved the protocol for research activities (protocol #2018682) with an expedited review to ensure compliance with ethical policies, in accordance with the principles of Helsinki. In addition, the Texas Department of State Health Services (DSHS) Institutional Review Board (18-029) deemed the study exempt for the full extent of the project. Each participant was emailed a USD 50 Amazon gift card as compensation for their time. 

### 2.2. Participants and Recruitment 

**Figure 1:**
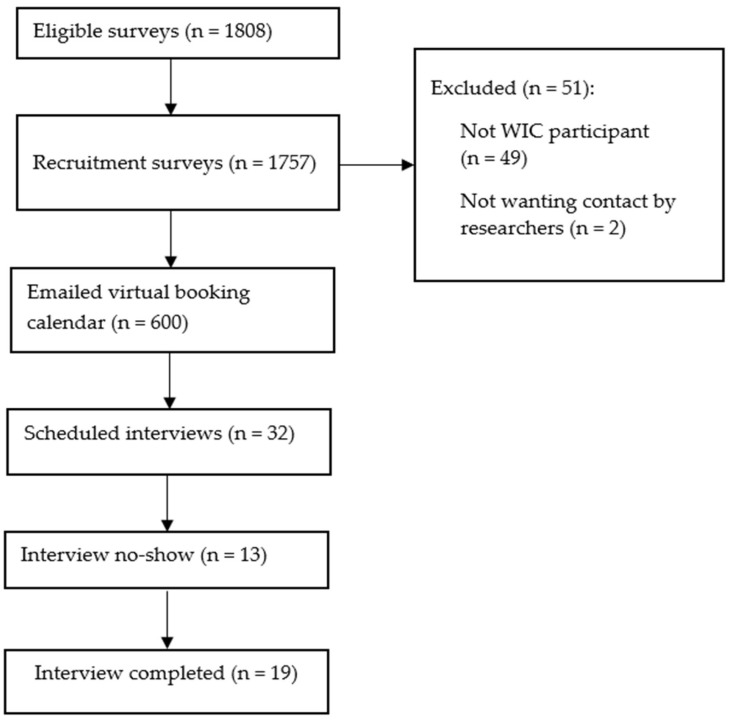
Recruitment of study participants (n = 19). WIC—the Special Supplemental Nutrition Program for Women, Infants, and Children.

This study recruited a convenience sample of current Texas WIC clients aged 18 years and older, who were interested in technology-mediated access to information. Through a collaborative effort with Texas WIC, the research team recruited participants through a targeted social media campaign over a 2-week period in June 2022. The online flyer provided a brief explanation of the research’s purpose and a hyperlink to an online screening survey. Recruitment was part of a larger evaluation, which can be found in the Chat with WIC Special Project Grants report [17]. Survey participants were excluded (n = 49) because they were not current Texas WIC clients, and because they did not want to be contacted by the research team (n = 2) (Figure 1).

### 2.3. Theoretical Framework 

**Figure 2:**
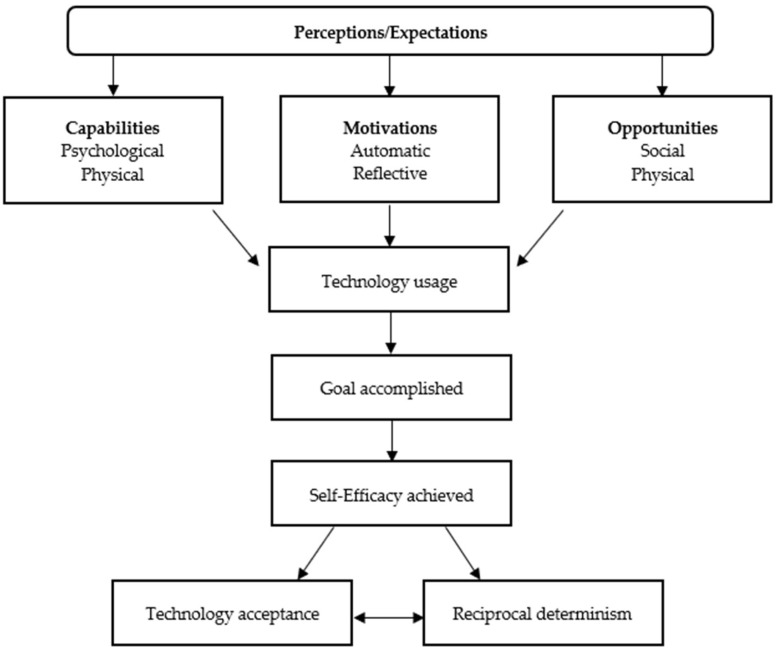
Combined theoretical framework model [42,43,44,45,46].

To the best of the authors’ knowledge, there is no existing framework that includes factors and behaviors (or user performance), chatbot performance, and the interaction of user performance with chatbot performance. This study created and utilized a combined theoretical framework (Figure 2). The Health Belief Model [43] and Social Cognitive Theory [44,45,46] were helpful for understanding how intra- and interpersonal perceptions, expectations and environments related to clients seeking nutrition information from a chatbot. The Capabilities, Opportunities, and Motivation Behavioral Model (COM-B) was utilized to explore the essential conditions required for participants to engage with the chatbot [47]. The Technology Acceptance Model was incorporated into the theoretical framework for the adoption and acceptability of receiving nutritional information through the chatbot [42]. This theoretical framework informed the interview guide, codebook development, and analysis (see Section 2.4,Section 2.5,Section 2.6). Table 1 identifies the key constructs of theories used.

Figure 2 depicts a combined theoretical framework utilizing constructs from the Health Belief Model [44], Social Cognitive Theory [45,46,47], Capabilities, Opportunities, Motivations, and Behavior change wheel [48], and Technology Acceptance model [40]. The arrows in the figure indicate a direction of influence from one construct or behavior to another.

### 2.4. Instruments 

In collaboration with Texas WIC, the research team developed a brief demographic survey to collect essential information about the sample and a semi-structured interview guide to explore the following five nutrition information topics related to infant feeding practices: (1) breastfeeding, (2) formula feeding, (3) feeding adequacy, (4) safety and troubleshooting, and (5) complementary feeding and first (solid) foods. Findings from previous evaluations showed that clients identified those topics as priorities for the content or delivery of nutritional information [17,36]. The research team used the theoretical framework to develop the interview guide and modified the interview guide based on initial observations of the interviews (Supplementary Table S1).

### 2.5. Data Collection and Management

The study team emailed the first 600 survey respondents and asked them to schedule an online interview via an electronic scheduling booking site. Clients scheduled online interviews through email and text messages. After the clients had booked interviews, they provided informed consent via Qualtrics online surveys [49]. Thirty-two clients booked interviews, and the study team completed 19 interviews until they determined that they had reached saturation. Frequent team discussions about data, memorandum (memo) writing, and coding were used to determine the saturation of themes [50] (see Data Collection and Data Analysis sections). This study was completed in English.

WIC program leadership aimed to minimize respondent burden and informed the decision to collect only the demographic characteristics needed for the study, such as age, education, and household composition. Each client completed a video interview on the Zoom platform; both the audio and video footage were recorded [51]. A moderator asked open-ended questions, utilizing the semi-structured interview guide (Supplementary Table S1). The interviews were designed to last 45–60 min. The interviews continued until saturation of themes was determined through a review of field notes, initial coding, and research team discussions. All interviews were conducted during July of 2022. A professional transcription company created interview transcripts from the audio recordings.

### 2.6. Data Analysis 

**Figure 3:**
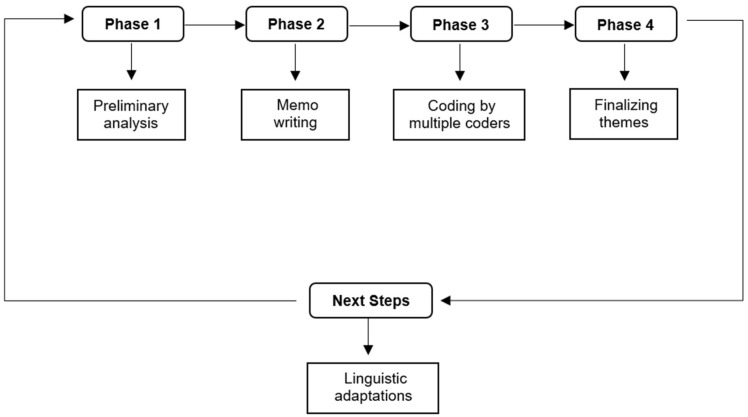
Multi-phase analysis based on user-centered design. This figure shows the different parts of the qualitative analysis based on user-centered design.

Quantitative data from sociodemographic surveys were exported into a Microsoft^®^ Excel spreadsheet for analysis. Qualitative data analysis was completed manually using transcripts. The transcripts were reviewed and coded incident-by-incident through multiple phases of coding (Figure 3). There were multiple coders involved in the analysis.

Phase 1 included preliminary analysis for Texas WIC reporting, as seen in Supplementary Material Table S2. The Phase 2 analysis involved memo writing and was initiated during data collection after interviews were conducted. During data collection, our research team met routinely to discuss observations about the interviews, and peer debriefing continued through coding [52]. In addition, this analysis utilized two rounds of coding of the interview transcripts. Memos were created throughout phase 2 to maximize transparency and facilitate an open coding process. The following paragraphs describe the phases of codebook development and rounds of coding in detail. 

During phase 3, the research team created a codebook based on the theoretical framework (Table 1), as shown in previous studies [52,53,54,55,56], and coded interview transcripts. The initial set of codes, called primary codes, were expanded to include secondary codes, which allow for specificity when coding incidents. For example, CAP was the primary code for discussing a client’s physical capabilities. Secondary codes of CAP-P and CAP-N were created to understand the context of the discussion related to physical capabilities and identify them as positive (CAP-P) or negative (CAP-N). Coders used the codebook while coding and made updates as needed. Coders wrote memos for new codes (to identify and justify the value of a new code and provide examples of how to apply that code), new incidents (that were identified at a later pass through), and discrepancies (to identify differences in coding or incident interpretations) [50,53]. See Supplemental Table S3 for additional examples. All coding revisions were agreed upon by the full research team. As codes were created, any previous interview coding and memos were reviewed and annotated to ensure cohesive analysis across all interviews [53]. 

A team of three coders completed coding of interview transcripts. Two coders completed incident-by-incident coding, and a third coder addressed and helped resolve discrepancies. Coding was completed in multiple passes. The first pass of coding was conducted to extract top-level findings to present to Texas WIC. For the second pass of coding, two coders conducted incident-by-incident coding with real-time peer debriefing [52,53,54,55,56,57,58]. Both coders wrote memos to identify new incidents, new codes, and discrepancies. During data analysis, the coding team used peer debriefing to discuss themes and coding discrepancies. The intercoder reliability, or percentage agreement in coding, was 84.9%, based on 662 text segments or passages with agreement and a total of 780 text segments (662 in agreement and 118 in disagreement). The intercoder reliability indicated strong reliability [58,59]. In addition, during the coding process, coders wrote advanced memos and posted them to interview transcripts (Supplemental Table S3) [50,53]. This process continued until saturation of codes was achieved and all interviews were reviewed with the saturated code list [54].

In Phase 4, to finalize the themes, all codes and memos were grouped together by similarity (Supplementary Table S4). Original interview transcripts were referenced, as necessary [54,60]. The research team identified, diagramed, and mapped codes and memos which identified three primary themes: motivations; capabilities; and opportunities [47,50,54]. Within each primary theme, codes and memos were further condensed into subthemes [50,61]. Saturation of themes and content was determined by the lead researcher and our research team through an iterative process [50,61]. All processes for initial and thematic coding were checked and rechecked throughout the analysis via triangulation [50,58]. Lastly, quantitative data from the sociodemographic survey were analyzed descriptively.

## 3. Results

### 3.1. Clients 

WIC program leadership determined which sample demographic characteristics were needed for the study to minimize respondent burden, and this study collected information about participants’ age, education, and household composition. Clients self-identified as current Texas WIC clients and mothers (n = 19). Ages ranged from 18 to 44 years, with an average of 26.7 years old. The most common education levels were a high school diploma/GED (7/19, 36.8%), some college (9/19, 47%), associate’s degree (2/19, 10.5%), and bachelor’s degree (1/19, 5.2%). Most clients (15/19, 78.9%) had two or more children that currently, or had previously, received WIC benefits. When asked how old their youngest child was, 14 clients (14/19, 73.6%) had a child of 1 year or younger, 3 clients (3/19, 15.8%) had a child aged between 2 and 5 years, and 2 clients (2/19, 10.5%) were currently pregnant. Some clients indicated prior use of Maya, the Texas WIC chatbot (4/19, 21.1%).

### 3.2. Nutrition Information 

**Figure 4:**
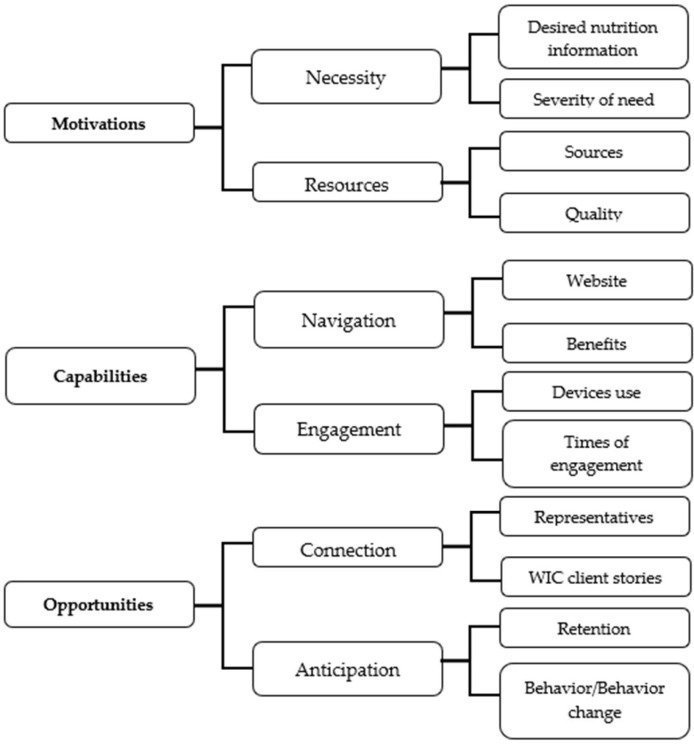
Flowchart for the themes and subthemes related to clients seeking nutrition information via a chatbot. This figure demonstrates the hierarchy of thematic findings. The primary themes identified were motivations, capabilities, and opportunities. The middle and right side of the figure refer to the secondary and tertiary themes.

Analyses generated three primary themes related to clients’ (1) motivations, (2) capabilities, and chatbot usability, and (3) opportunities for seeking nutritional information via the chatbot. Motivations were influenced by necessity and resources. The usability of the chatbot was important for navigation and engagement. The chatbot offered opportunities for connection and some beneficial assumptions. Figure 4 shows a flow chart of the themes and subthemes.

### 3.3. Motivations 

Clients identified the necessity of information and resource availability as key motivators for seeking nutritional information through Maya (Supplementary Tables S5 and S6).

#### 3.3.1. Necessity 

According to this sample of clients, gaining nutrition information through Maya was necessary to learn updates regarding benefit package changes and understand program adaptations to crisis (i.e., COVID protocols, COVID package changes, formula crisis).

*“I check (TexasWIC.org) every 2 to 3 days, because I like the updates with the formula and stuff as in my daughter’s formula got switched. So, I like to sit there and see if (there are) any alternatives and things like that.”*

*(Client 5)*

Table 2 shows a comprehensive list of topics, desired content, potential for the chatbot, with quotes. Clients identified the following topics as most important: benefits, troubleshooting breastfeeding issues, complementary feeding how-tos, picky eating, baby signs and cues, recipes, shopping assistance, online classes, WIC program and package updates, healthy eating practices, hazards (i.e., choking, food allergies), and connecting to other assistance programs, such as the Supplemental Nutrition Assistance Program (SNAP).

*“I have no idea how to cook vegetables the right way…. (If Maya could help with) how to prepare these things it would be super helpful, because some of us just were not raised in healthy households.”*

*(Client 18)*

#### 3.3.2. Resources

Clients expressed motivation to obtain nutrition information through electronic WIC resources (i.e., texasWIC.org, MyTexasWIC mobile application, Texas WIC online classes, WIC SMS) and external electronic resources (i.e., search engines, nutritionist websites, and social media.) Many clients identified accessing the MyTexasWIC mobile application and WIC online classes through their mobile device as their most consistent and preferred method of obtaining the desired information (i.e., benefit understanding, shopping assistance, and recipes). They accessed information at different times for different purposes, such as pre-planning shopping, seeking real-time help, or looking up questions after something occurred. 

Clients discussed trustworthiness and efficiency of time as motivators for engaging with an electronic resource. Trustworthiness was said to have been achieved via external WIC resources after being vetted for reliability by (1) confirming professional recommendation(s) received; (2) validating or normalizing their individual experiences; or (3) if authors claimed to be nutritionists or dietitians. Clients described the vetting process as less desirable because of the additional time required to ensure trustworthiness. WIC resources were discussed as the most desirable due to expressed trustworthiness in the program and in staff expertise in nutritional and client benefits specifically.

*“(I need nutrition information from Maya because) I trust the WIC program. They know the nutrition values that the kids should have and the alternatives.”*

*(Client 11)*

Maya was discussed as an efficient method to obtain information more quickly than it could be obtained through other standardized WIC practices (i.e., visiting a clinic, calling the hotline, or emailing) or through external resources. Efficiency was also identified, as responses were provided in real time, were available quickly, were presented concisely, and could be customized at any time of day.

*“I think that (Maya) will help by making information readily available. Not having to wait until WIC office opens up. Especially if you have a question that is after hours or on the weekend.”*

*(Client 13)*

### 3.4. Capabilities of Clients and Usability of Maya 

In the recruitment survey, approximately one-third of clients initially reported that they were unlikely to seek nutritional information from a chatbot. In interviews, clients stated that poor prior experiences with any chatbot influenced their perceptions regarding any chatbot’s ability to successfully complete a task. Poor prior chatbot experiences, not specifically with Maya, were attributed to the chatbot’s performance despite the clients’ physical and psychological capabilities to interact with the chatbot. All interview clients expressed a desire to receive nutritional information through Maya after receiving chatbot demonstrations. Clients identified Maya’s usability as a way of finding necessary information. Furthermore, clients discussed the conditions in which they would seek nutritional information through Maya and how they preferred Maya to perform, and their anticipated interactions with Maya. 

#### 3.4.1. Navigation 

Clients identified Maya as a navigation tool for TexasWIC.org and an informational tool for their benefits. As displayed in Table 2, clients stated that Maya could help them navigate resources by providing information in the form of visual aids (i.e., online classes, videos, diagrams), hyperlinks to webpages and online classes, and Supplementary Information (i.e., synopsis of webpages, fun facts, and counseling handouts).

*“I think that the WIC Chatbot can make it easier to find information versus having to go search on the WIC website yourself…She could give you more information than you could get off a webpage on the WIC website.”*

*(Client 14)*

Clients stated that Maya increased their psychological capability to understand their benefits (i.e., shopping education, explanations of package content or changes) and use more of their benefits package (i.e., customed recommendations and access to various recipes).

*“Using WIC is more consistent (than using search engines) because they know what kind of ingredients I already have; so that is a big one up on helping me find recipes that I can utilize my benefits with.”*

*(Client 3)*

#### 3.4.2. Engagement 

Clients identified devices and times in which they would engage or disengage with a chatbot. Most clients identified their phones as the primary device that they used to seek nutritional information electronically. Clients expressed a desire for Maya to be available via the MyTexasWIC mobile application for three reasons: (1) the MyTexasWIC mobile application was their preferred source of electronic WIC information; (2) to ensure the greatest compatibility with their mobile devices; (3) the MyTexasWIC mobile application automatically opened when accessing the texasWIC.org website on a mobile device.

*“I would use the app (to obtain nutrition information). If Maya is on the app- That would be really convenient because the app is my go to.”*

*(Client 12)*

Participants’ reasons for seeking nutrition information electronically included satisfying WIC nutrition education requirements, troubleshooting, or answering questions they had regarding a variety of topics. Some clients speculated that Maya would be particularly helpful to first-time mothers and parents of children with large age gaps. Clients stated they would engage with Maya when planning, troubleshooting, shopping, and when they had a question.

*“I think (Maya troubleshooting breastfeeding issues) would be a need because I remember struggling to produce. So, I wasn’t sure, like should I be eating something specifically, what can help me with more milk supply? So, I think when I was at that point it was like a desperate mommy moment, and I would say I would need it.”*

*(Client 9)*

Some clients stated they would stop using the chatbot if the device’s screen size was not compatible for tasks or placed large demands on device storage/memory or their device’s battery, or if they lacked access while shopping. Inefficient chatbot performance (i.e., providing vague, indirect, or lengthy responses to a question) was mentioned as another reason to disengage with Maya. A few clients also stated they would not use Maya if it provided information that promoted behavior contrary to their own personal practices.

*“(It would not be helpful if Maya was) very aggressive in recommending (breastfeeding)…That wouldn’t make me want to go to the site much or use the bot at all.”*

*(Client 3)*

### 3.5. Maya Opportunities 

Most clients discussed opportunities for Maya to foster connections as well as provide supportive responses. 

#### 3.5.1. Connections 

Clients consistently discussed a desire for support from live representatives such as WIC staff, nutritionists, and lactation consultants during their interactions with Maya. Clients differed in their opinions regarding when to connect to a representative. Some clients wanted an immediate connection with live representatives; others wanted to be connected with live representatives after first receiving top-level information.

*“I know that WIC has lactation specialists, but it would be nice to just log on the website and see ‘this will help produce milk’ or ‘try this’ or maybe some sources on there.”*

*(Client 6)*

Clients stated they had previously sought out various electronic sources (online platforms, social media groups, and internet searches) to obtain information and support regarding infant or child feeding. Statements of trustworthiness were mentioned if the stories felt authentic and relatable.

*“(Stories felt reliable because) they were from other people who had experienced the same kind of situation and it made more sense that we weren’t the only one having the issue, there were other people. It was normal.”*

*(Client 7)*

#### 3.5.2. Anticipations 

Clients discussed their desires for Maya to provide connections to WIC representatives and access to WIC client stories; to promote behaviors (i.e., continued breastfeeding, healthy eating, and maximize benefit utilization); and facilitate behavior change (i.e., increase fruit and vegetable intake and address picky eating).

*“How to actually prepare (vegetables and healthy foods) would be super helpful, because some of us just weren’t raised in healthy households.”*

*(Client 18)*

Some clients stated that if they were met with encouragement from Maya, it could provide reassurance and motivation to continue breastfeeding during times of difficulty (i.e., clogged ducts, latching pain, cluster feeding).

*“I was seeking mommy troubles that you come across with breastfeeding. That sometimes you may get- like a clogged duct; or something that would tell me how that could be fixed- or just to keep me going so I’m not discouraged from breastfeeding. Because I know that when I had that issue- I mean- it was pretty painful and I really contemplated (breastfeeding), because it hurt.”*

*(Client 9)*

Clients identified Maya as a potential resource for utilizing and retaining benefits, which (1) connected them to required online classes, (2) provided explanations of how to use their benefits (i.e., where, and how to shop), and (3) communicated customized feedback to help them retain their benefits at times of transition (i.e., suggestions about online classes, explanations of package changes, alerts in the event of operation changes).

*“This is my first time hearing about Maya. I just got back on WIC a couple of months ago. I had a pause to it because COVID offices were down; so, it is kind of difficult to get your WIC. So, this is pretty convenient (for getting my benefits).”*

*(Client 8)*

## 4. Discussion

WIC clients who participated in this study were motivated to seek nutrition information utilizing Maya, the chatbot, once they were familiar with the chatbot and understood its capabilities. They also expressed a preference for using the chatbot compared to other modes of live communication, such as calling a hotline or going to a WIC office. The findings are consistent with previous studies which demonstrated that chatbots were favorable and continually used when responses were human-like [28,62,63], supportive [24,62,63,64] and perceived to be personalized [62]. Future iterations of Maya could facilitate providing nutrition information to clients and could possibly deliver nutrition information via online nutrition education. In addition, WIC clients described preferences for customized interactions. The findings are consistent with published research showing the benefits of technologies created with a UCD approach for clients of WIC and SNAP-Ed programs [15,65].

Trustworthiness and efficiency of communication are essential to clients chatbot experiences, satisfaction, and repeated use [64,66]. In this study, clients identified that they were motivated to engage with Maya because they trusted the WIC program to deliver useful, relevant, and accurate information. The clients in our study identified Maya as an efficient facilitator of knowledge and information. This study’s findings suggested that Maya is desirable by its users to be capable of facilitating the delivery of nutrition information. This finding is consistent with a study by Greenblatt et al., which aimed to understand the experiences of Arizona WIC staff and clients when it came to providing and accessing nutrition information. Researchers identified that clinic constraints, such as the need to engage children while parents engage in consultations, influenced client’s receptiveness to receiving nutritional information [67]. The findings of Greenblatt at al. promoted technology-based approaches including quick facts, tips and tricks, and video and interactive modules over handouts and counseling tools, which were perceived as “time-consuming and unresponsive to client preferences” [67]. Contrary to the findings in our study, Munnukka et al.’s study investigated virtual chatbots and determined that the acceptability, social presence, and trust of recommendations delivered from a virtual chatbot could be related to longer and more human-like responses [62]. The differences in these findings could be a result of different population needs, different roles between the two different organizational interfaces, and differences in the type of chatbots (i.e., AI vs. dialog-based).

The clients in this study discussed the need for Maya to introduce itself and explained how Maya can support client engagement. Several clients also identified a need for Maya to be available as an independent mobile application or on the current MyTexasWIC app. Chatbot availability across multiple platforms is supported by a scoping review of 23 lifestyle chatbot studies across the globe, which concluded that chatbots may be most effective in promoting lifestyle changes when integrated with other technologies to encourage and support behaviors, according to Chew, HSJ et al. [28].

Capability was a major theme in our study; clients self-identified as capable of using Maya to obtain nutritional information. Subsequently, clients discussed chatbot usability as a factor in their capability to efficiently navigate TexasWIC.org, online classes, and the use of their benefits. Some clients stated that receiving nutrition information via Maya may impact their behaviors, resulting in them maximizing their benefits, eating more fruits and vegetables, and continuing with the WIC program. The current literature is unclear as to whether nutritional information provided through a chatbot would promote behavior change or program retention [29,63,68,69].

In our study, clients expressed strong desires for nutritional information to be available through Maya, with breastfeeding, formula feeding, and complimentary feeding listed as the most important topics. Our study clients expressed desires for Maya to facilitate connections to encouragement, other client stories, and various WIC representatives. Maya has the potential to connect WIC clients to information and support in times of difficulty, such as pain while breastfeeding or beginning complementary feeding (client-identified needs). The existing literature supports our findings that encouragement [35,70] and story sharing are desired technology features [70,71,72]. However, it is important to moderate stories and content [71,72]. Future research could investigate the importance of moderated peer story-sharing and chatbot encouragement, as well as their impact on client behaviors in relation to WIC program outcomes.

### 4.1. Strengths and Limitations

Collaborations with the Texas WIC state agency office was a strength of this study. An additional strength of this research was sampling through an online platform, which allowed for a larger recruitment pool across the state. The research team was an independent group, not part of Texas WIC, which enabled this study to minimize social desirability bias. For example, clients were able to freely share their perspectives, knowing that their WIC benefits would not be impacted by anything they said or did in the interviews. Lastly, the study applied rigorous techniques for data analysis and interpretation to ensure the findings were reliable and valid.

A limitation of this study, as with all qualitative studies, is that the findings may not be generalizable to the WIC in other states, Indian tribal organizations, or territories. Furthermore, perceptions, trustworthiness, and engagement with a chatbot can vary based on cultural expectations [26,61,65,68]. This study, as an initial effort, focused on clients of the English version of Maya and did not include the Spanish version. Over half of the Texas WIC client population identifies as Hispanic or Latino/a, and 36% of this population prefers to speak in Spanish [3]. Prior studies have found that expectations and utilization may differ between English and Spanish clients [65,67,68]. Future research is warranted to explore desires and potential cultural influences among Spanish Maya clients. A cultural adaptation may focus on preferences in chatbot engagement or the delivery or focus of nutritional education [15,62,65,67,68]. Future studies will need to consider which demographic variables related to race, ethnicity, or culture, including language, are essential for interpretation given the drawbacks). It is also important to conduct additional research to assess how this chatbot can achieve a greater impact by reaching a larger population of clients or different client subgroups with diverse needs.

### 4.2. Implications for Research and Practice

This study indicates that WIC clients may view Maya as a catalyst for accessing trustworthy nutrition information, including in times of distress (i.e., breastfeeding difficulties, picky eating), program adaptations (i.e., pandemic, formula crisis, The Child Nutrition Reauthorization Act), or package updates (i.e., child maturation, COVID temporary fruit and vegetable vouchers) [73]. The findings may inform programmatic changes that improve WIC participation and alleviate the administrative burdens of local offices. Though this study was aligned with WIC, other public health programs may be able to develop and test a chatbot to provide information and evaluate the impacts. Future considerations relate to trustworthiness in chatbot development and modification. For example, if chatbots are used to provide nutrition information, then programs will benefit from transparency in how responses are generated. Further, as technology progresses, chatbots are being seen as opportunities within business, communication platforms, and public health [18,19]. Though they have limitations, these chatbots hold promise, as studies have reported they are efficient and inexpensive to change, while the present study demonstrated interest in several complex topics [18,19]. Regarding research, future studies are needed to test different ways to provide nutritional information about infant feeding via a chatbot. Additional research is also warranted to assess how quickly and accurately a chatbot responds, as well as the long-term impact of utilizing Maya. For example, longitudinal studies involving this chatbot can assess changes in Texas WIC clients’ nutrition knowledge, behavior, and program participation, which would be beneficial for understanding its effectiveness.

This study utilized a dialog-based chatbot with predefined responses and rules devised by human developers. While this allows for controlled, predictable responses to client prompts, there is growing interest in moving from dialog-based chatbots to more advanced AI chatbots based on machine learning algorithms. Other public agencies, global organizations, and public health entities may choose to use this study’s framework and the UCD approach to identify, develop, and evaluate new chatbots. Additionally, it may be beneficial to explore whether more advanced AI chatbots can improve user experience and outcomes compared to dialog-based chatbots. Testing different response-generation approaches and analyzing conversational data over time may shed light on ideal mechanisms. 

## 5. Conclusions 

In conclusion, WIC clients expressed motivations, capabilities, and opportunities to engage with nutrition information through an existing dialog-based chatbot. The methodology used in this study represents one innovative approach to “give participants user-centered choices for how they participate in WIC” (p. 21) [13]. The findings of this study highlight the potential impact of technology-based innovations on Texas WIC participation and utilization of benefits. Future WIC technology innovations can benefit by exploring users’ expectations to obtain nutrition information through WIC technologies. Further public health research may indicate if this methodology and other technologies, such as mobile applications, are preferred by some clients as sources of urgent information or support outside of standard operating hours. Future studies may utilize the theoretical framework or findings to advance m-health interventions within existing public health programs and help interventions achieve a greater impact.

## Figures and Tables

**Table 1:** Definition of codes grouped by theory and theoretical construct.

Theory	Constructs	Codes	Code Definitions
TAM [42]	Technology perceptions and usability	PU (Perceived usefulness)	Descriptive to beliefs and motives surrounding the use of technology and its ability to benefit the user.
		EU (Ease of use)	Perceptions, beliefs, and motives surrounding the ability to utilize technology effortlessly and without barriers.
HBM [43] SCT [44,45,46]	Capabilities (beliefs and physical)	SE (Self-efficacy)	The perceived ability to engage in a desired behavior to achieve a goal.
COM-B [47]	Capabilities	CAP (Physical)	Personal abilities and environmental access required to engage in desired behavior.
		CAPSY (Psychological)	Beliefs and/or knowledge that allow someone to engage in desired behavior.
	Opportunities	OS (Social)	Social support and/or environmental tools that are needed to achieve behavior.
		OP (Physical)	Physical access to tools and environments that are needed to achieve one’s desire.
	Motivations	MR (Reflective)	An intentional (planned and/or evaluated) drive in response to a stimulus or behavior.
		MA (Automatic)	An innate and/or associative disposition that may be emotional or impulsive that incites or is influenced by behavior.
HBM [43]	Perceptions	PB (Perceived barrier)	Beliefs about difficulties or inaccessibility that directly affect engagement of desired behavior.
		PS (Perceived severity)	Beliefs about how something directly impacts one’s ability to engage in desired behavior.
SCT [43,44,45,46,48,49,50] COM-B [47]	Behavior engagement	RD (Reciprocal determinism)	Behavior (tech use/seeking nutritional information) driven by personal factors (COM/perceptions/expectations) and environment (physical, social, or technological).
SCT [44,45,46]	Expectations	EOI (Expectation of information)	Anticipation, belief, or desire regarding the information received or that one wishes to receive.
		EOT (Expectation of technology)	Anticipation, belief, or desire of a technological performance.
SCT [44,45,46]	Desired nutrition information	DNI (Desired nutrition information)	Coding structure developed to identify thematic responses of investigatory topics that were informed through concurrent chatbot research, including Frequently Asked Questions analysis, survey responses, and relevant forum analysis.
		DNIDM (Desired nutrition information delivery method)	
Emergent	Emerging Theme	ET (Emergent theme)	Discussion points that were unanticipated.

Abbreviations: TAM—Technology Acceptance Model [48]; HBM—Health Belief Model [43]; SCT—Social Cognitive Theory [44,45,46]; COM-B—Capabilities, Opportunities, Motivations, and Behavioral theory [47].

**Table 2:** Key interview findings about content, potential suggestions for a chatbot, and relevant quotes organized by infant feeding topic.

Topic	Content Desired	Potential for Chatbot	Quotes
Breastfeeding * All of clients	Milk supply Pumping Partial BF	Online Classes and Visual aids Encouragement Live representatives Engagement	“I know that WIC has lactation specialists, but it would be nice to just log on the website and see ‘this will help produce milk’ or ‘try this’ or maybe some sources on there.” (Client 6)
Formula feeding * More than half of clients	Benefits Safety Guidance Gas/Colic Other	Online Classes and Visual aids Responses Navigation Emergent	“I would be okay with (general information and direction to a WIC nutritionist/pediatrician). I would be fine with it. Like if (Maya) weren’t able to give me the information I needed, at least guide me in some sort of direction.” (Client 9)
Adequacy of infant feeding * More than half of clients	How tos Signs/cues Other: Connect to HHS programs.	Visual Aids Responses	“I think (Maya) could make it easier by explaining output. If your baby is having X number of dirty diapers than you know they are getting enough.” (Client 14)
Safety and troubleshooting of infant feeding * More than of half of clients	*Feeding* *Pumping* *Hazard (choking and allergies* *Cleanliness*	Visual Aids Responses	“I think that (formula bottle safety webpage) is awesome, and I kind of wish if they have something like that with breastfeeding, as far as how to clean the pumps, how to store.” (Client 18)
Complementary feeding * *More than half of clients*	How tos Eating styles Preparation Others	Online classes/Visual aids Responses Engagement	“(I would like to) Search up ‘videos on baby’s first foods’, that helps.” (Client 9) “I would so love Maya to have some recipes and see ideas of what to give my baby next.” (Client 6)
Emergent Themes			
Benefits * Some of clients	Awareness Other	Online Classes Responses Engagement	“(Benefits look up) would be super helpful. Since the app takes up too much storage, being able to go on the website and look it up super easily, that would be very helpful. (Client 18) “Maya can give you a better scale of approved fruit and vegetables that are approved, fresh frozen, or even jarred.” (Client 5)
Recipes	Directory of recipes	Online Classes and Videos	“I can see (recipes on Maya) being beneficial not only for me, but other families as well.” (Client 7)

* Indication of number of clients that provided feedback about specified topics. Abbreviations: BF—breastfeeding; HHS—health and human services; WIC—the Special Supplemental Nutrition Program for Women, Infants, and Children.

## Data Availability

The original contributions presented in this study are included in the article/Supplementary Materials. Further inquiries can be directed to the corresponding author.

## Supplementary Materials

The following supporting information can be downloaded at https://www.mdpi.com/article/10.3390/ijerph22020193/s1: Table S1: Interview flow and description of activities; Supplementary Materials Table S2: Initial findings report; Table S3: Thematizing memo topic: accessing Maya; Table S4: Thematizing memo topic: nutrition information; Table S5: Thematizing memo topic: health belief model constructs; Table S6: Comprehensive interview findings by investigated topic: motivation.
