# Supplementary material for: Exploring Client Perceptions on Gaining Infant Feeding Information Through the Texas Women, Infants, and Children (WIC) Chatbot
Source: Int J Environ Res Public Health. 2025 Jan 29;22(2):193. doi: 10.3390/ijerph22020193 (PMC11855084; doi:10.3390/ijerph22020193)
Supplement: Supplementary file 1 [file ijerph-22-00193-s001.zip › Supplemental Material Table S2.pdf]

Objectives

Our primary objective was to interview Texas WIC clients to explore their interest and preferences for potential infant feeding education that could be accessed through Maya, the Texas WIC chatbot. A secondary objective was continue evaluating Maya's general usability and alignment of functionality with user expectations.

Key findings and recommendations (Appendices: 1 Nutrition topics, 2, Top level findings)

Primary objective

Current sources for nutrition education included Texas WIC, internet searches, social media, nutritionist websites, and family members. Participants mentioned picky eating and finding recipes as important motivators for seeking nutrition education. Many participants preferred Texas WIC as a source of nutrition information because it was trustworthy and centralized (i.e., a 'one stop shop'). Participants' trust in Texas WIC extended to potential nutrition information from Maya and believed it be a useful way to find nutrition information, and that it would be a feature they would want to use. The Maya nutrition experience should was also desired to provide customized interactions. (e.g. know their name).

Table 1. Key recommendations for developing nutrition education content for Maya.

Desired nutrition education topics	Desired mode of delivery
• Recipes, searchable by ingredients<br>• How to introduce first foods<br>• Signs of readiness for food<br>• Breastfeeding support<br>• Selecting and troubleshooting bottles<br>• Identifying gagging and choking<br>• Info on mixed feeding (breastmilk / formula)<br>• Navigating feeding transitions (weaning, formula to exclusively breastfeeding, mixed feeding, switching formulas, switching to/types of milk)	• Videos<br>• Links to online classes<br>• Personalized recommendations appropriate for their child's age, their interests, and required nutrition education<br>• Breastfeeding resources, especially helping with latch<br>• WIC-led or -approved social media groups and forums<br>• Links to relevant TexasWIC.org web pages<br>• Short summaries of TexasWIC.org pages<br>• Referrals to appropriate WIC and outside professionals<br>• Downloadable resources<br>• Affirmations and caregiver stories

Secondary objective

Participants who had used Maya previously (n=4) rated their previous experiences as helpful and useful
Participants who had not used Maya (n=15) cited unfamiliarity due to: a preference for using the MyTexasWIC app instead of TexasWIC.org, poor prior experiences with other chatbots, and being unaware of Maya's capabilities, purpose, and/or location.

Table 2. Key recommendations for improving the usability and functionality of Maya.

Increase Maya awareness	Feature suggestions
• Increase visibility on small screens by enlarging the Maya icon or locating near the search bar<br>• Add animations to chatbot button (e.g., button enlarges or dances)<br>• Maya pops up to initiate chat<br>• Info about Maya on the MyTexasWIC app	• Allow users to communicate with WIC clinics via Maya<br>• Personalization (e.g., greeting by name, providing recommendations based on their benefit packages)<br>• Help users understand and navigate benefits packages<br>• Additional dialogs providing detailed shopping help

Participant Characteristics (Appendices: 3 NI Recruitment Survey; 4 NI Respondent Results)

- All participants were mothers and active Texas WIC client, ranging in age from 18 to 44 years.
- Education: High school diploma/GED (37%); Some college (47%); Associates (11%); Bachelors (5%)
- Age of youngest child: 1 year old or younger (n=14); 2-5 years (n=3); pregnant (n=2).
- Most participants (n=15) had two or more children currently or previously enrolled in WIC.

Methodology (Appendices: (5 NI Guide; 6 NI Slides, 7 Master Findings sheet)

Participants were recruited through Facebook advertisements targeting current Texas WIC clients, which linked to an online screening survey. Respondents who were current Texas WIC clients (n=1,706) were invited to schedule an interview. Semi-structured interviews (n=19) were held online. Interview participants were provided with a $50 Amazon gift card.
