# Supplementary material for: Exploring Client Perceptions on Gaining Infant Feeding Information Through the Texas Women, Infants, and Children (WIC) Chatbot
Source: Int J Environ Res Public Health. 2025 Jan 29;22(2):193. doi: 10.3390/ijerph22020193 (PMC11855084; doi:10.3390/ijerph22020193)
Supplement: Supplementary file 1 [file ijerph-22-00193-s001.zip › Supplementary Table S1.pdf]

Supplementary Table S1. Interview flow and description of activities.

Activity	Description of Activity
Emoji Activity	A chart of varied emojis was displayed. Participants were asked to answer a series of questions with the most appropriate emoji. of questions were asked. Participants may be asked to elaborate why they chose their emojis.
Maya Demonstrations	Demonstrations of the Texas WIC chatbot were provided to participants that indicated they are unfamiliar with Maya or chatbots in general.
Flowchart activity	A diagram of the WIC flow of services was displayed. Open ended questions explored opportunities and motivations to use the chatbot for nutrition information.
Ranking activity	Collected participant feedback throughout the interview was repeated back to the participant. Participants were then asked to rank the importance of their feedback as a must have (need), would like (want), or indifferent.
WIC Website Demonstrations	Nutrition content from TexasWIC.org was displayed to participant's to explore the client's perspective of the chatbots topic.
