# Supplementary material for: Exploring Client Perceptions on Gaining Infant Feeding Information Through the Texas Women, Infants, and Children (WIC) Chatbot
Source: Int J Environ Res Public Health. 2025 Jan 29;22(2):193. doi: 10.3390/ijerph22020193 (PMC11855084; doi:10.3390/ijerph22020193)
Supplement: Supplementary file 1 [file ijerph-22-00193-s001.zip › Supplementary Table S3.pdf]

Supplemental Table S3. Thematizing Memo Topic: Accessing Maya.

Current WIC Tech Trends	Current Trends	Expectations	Motivation
Phones are preferred due to convenience, portable, quick, no perceived barriers and few limitations.	Participants don't use websites unless it's for online classes or looking for updates.	Uses Maya for simple questions and ses website for complex questions [P5]<br><br>Negative beliefs: poor performance, misunderstood inputs, repetitive, and time consuming.<br><br>Positive beliefs: time saving [P12], avoids speaking with live representative, easier than calling [P10].	Severity: Many felt they don't get enough nutrition education therefore have to search on their own.<br><br>Consequences of pandemic<br><br>Going back in person [visits]<br><br>Build tech capacity
Using Texas WIC website for online classes, recipes, benefit utilization, crisis information.<br><br>Positive sentiments about the site.	Participants seek information related to shopping and preparing foods.	EOT: Wants Maya to access information fast, navigate website, access account information/benefits, understand updates, want "pop ups" [P3], call back feature, and role throughout WIC flow of services, navigate to specific section of website page.<br><br>Wants voice-to-text option and real time solutions/help.	Not seeking information when participant has previous children.
App: high favorability mostly using app for online classes, benefit updates, and shopping aid.<br>Routed to app automatically when on phone.<br>[P15] had storage issues on phones and disables phone functions on app setting.	Most not using Maya due to page layout, didn't know what it was, poor experiences with other chatbots, Maya isn't as noticeable if accessing the Texas WIC website on a mobile device, and question typos.	EOI: just a little information (doesn't want too much feedback from Maya), customized based on child's age, guides benefit updates and usage, promote and influence healthy cooking and eating, and should answer series of questions [P7]	Intention to understand benefits, meal plan and prepare for shopping.
Most were satisfied with the website and app but wanted Maya to be available on both.	After the demonstration of Maya, most participants stated excitement and intentions to use.<br>Motivation to use is based on beliefs of quick access to desired information.		Access to Maya after hours.
Chatbot: Some negative motivations to chatbots (viewed as limited, unhelpful in terms of connecting to a live representative).	Those who have used Maya previously indicated positive experiences with it.		Would use Maya to access information about formula crises, understanding changes due to crises (some participants checked the website daily for this information).

			Promote behavioral changes<br>[P3] Accessing recipes
			Used Maya: preemptively for meal planning, retroactively real-time for breastfeeding trouble, latching and crisis updates.<br><br>Seeking information during pregnancy or after birth.<br><br>Review website, then Maya, then call.<br>Chatbot avoids interactions with suspected perceived bias.

[1]

[1] *P indicates participants' number.
Abbreviations: WIC: The Special Supplemental Nutrition Program for Women, Infants, and Children. EOT: expectation of technology. EOI: expectation of information.
