# Supplementary material for: Exploring Client Perceptions on Gaining Infant Feeding Information Through the Texas Women, Infants, and Children (WIC) Chatbot
Source: Int J Environ Res Public Health. 2025 Jan 29;22(2):193. doi: 10.3390/ijerph22020193 (PMC11855084; doi:10.3390/ijerph22020193)
Supplement: Supplementary file 1 [file ijerph-22-00193-s001.zip › Supplementary Table S4.pdf]

Supplemental Table S4. Thematizing Memo Topic: Nutrition Information.

Current Trends	Desired Information	Desired Delivery
Participants are using technology to answer nutrition questions (picky eating, recipes, and complementary feeding).	Adequate feeding: feeding sleepy babies, hunger cues, fussiness, hydration during the summer, and diaper output.	What Maya as a phone app or on the current app.
Often site seeking stores [P6] (Like a Facebook mom groups, confirming normalcy, beliefs, and camaraderie.	Breastfeeding: leaking, clogged ducts, let down techniques, cluster feeding and timeline for anticipated clustering, latching methods, maternal diet for milk supply (enrichment and boosting)<br><br>Pumping: How, timing, cleaning, storage, thawing, flange sizes, getting pump, transition from/ to formula and or food.	Hyperlinks (external and internal) (i.e. connection to other programs, SNAP, Medicare).
Googling, nutrition websites, statements of trustworthy but difficult, some find empowering/ getting the real information.	Benefit understanding, recipes, availability, brands, changes to benefits, shopping recommendations, updates to benefits, appointment look ups [P18].	Page summary in chatbot, navigate to specific points on a page (not just to the page).
Wants Maya to give nutrition information	Formula: amounts by age, best bottles, water types, safe bottle warming, storage, switching types, bottle prep, remaining benefit availability, mixed formula type in same bottle, prevent/mitigate gassiness.	Videos directly in chatbot.
Wants to understand nutrition information's impact on health [P11].	Safety: Often discussed in breastfeeding, formula, separate safety information for breastfeeding and formula options, when seeking professional help, allergies, choking hazards, safe soaps, fruits and vegetable prep.	How To: complimentary feeding, breastfeeding, latching, pumping, MyPlate, cues and understandings.
Used txwic.org for benefits and formula update.	Complementary feeding:<br><br>Introduction to brands, allergen foods, reasons for spitting food out, safety prep methods, transition on/off cow's milk, stool changes, when to start, recipes, food idea's	Recipes: benefit maximization, exploring new foods, avoiding picky eating, and meal recommendations.

Accessing WIC online classes: Most participants mention the classes are helpful, but some do not view it as nutrition education.	Appointments: schedule, communication directly with clients/live representatives, benefit look-up, and reminders.	Visual aids: videos, PowerPoint slides, diagrams, live cooking classes.
	Recipes, benefit understanding and updates, complimentary feedings, preventing picky eating, help with food diversity, gestational diabetes mellitus information, specific recipes, healthy sways, different meal prep methods based on ingredients, age specifics, cultural inclusivity, and snacks, fruit/vegetables specifics.	Online classes: Maya hyperlinks to page, Maya suggests based off benefits received, and provides supplemental information, and summary of webpage content.
	Needs a mixed feeding option (breastfeeding and formula use), participant will disengage if prompted with opposite topic when searing [P3]. Bottle prep for formula, appropriate mixing, and needs how to suggestions.	Fun facts: nutritional values, tips and tricks (breastfeeding, gas) prep methods/ food activities, suggestion box.<br><br>Customized information based on package and based on child age [P5, P8].

[1]*P indicates participants' number.

Abbreviations: WIC: The Special Supplemental Nutrition Program for Women, Infants, and Children. EOT: expectation of technology. EOI: expectation of information.
