# Supplementary material for: Exploring Client Perceptions on Gaining Infant Feeding Information Through the Texas Women, Infants, and Children (WIC) Chatbot
Source: Int J Environ Res Public Health. 2025 Jan 29;22(2):193. doi: 10.3390/ijerph22020193 (PMC11855084; doi:10.3390/ijerph22020193)
Supplement: Supplementary file 1 [file ijerph-22-00193-s001.zip › Supplementary Table S5.pdf]

Supplemental Table S5: Thematizing Memo Topic: Health Belief Model Constructs.

Self-Efficacy	[1]Reciprocal Determinism	Barriers
Maya could help with understanding benefits and accessing WIC resources [P7].	WIC is a trusted resource for nutrition information.	Prefers to call instead of using Maya. If they used a chatbot then they would not trust the information [P8].
Maya could have helped with looking up benefits so the participant could have used them sooner.	Participants want to get their information first from Maya (any chatbot) quickly [P3].	Prefers nutrition education 1:1 because it feels like online is not nutrition education. Would use Maya if necessary [P9].
Lost benefits during pandemic and feels like Maya could have mitigated this disruption [P8].	WIC can give specific information about benefits and package utilization that other resources cannot provide (accuracy). Participants don't trust non-WIC sources about WIC information [P19].	Formula shortage: couldn't reach the office for a formula question. Resulted in not buying formulas, caused stress and anxiety for child's wellbeing [P17].
Some level of lactation information/ troubleshooting available without need to connect to a live representative [P6].	Participants are currently checking online classes for nutrition information first (informative and trustworthy).	Phone apps have limitations (storage, battery problems, data demands).
Seeking validation, early weaning, stories, determining trustworthy sources [P7].<br><br>Using 'social support' rather than validation.	Must be quick with responses or participant may leave site and google information [P3].	Limited access to pumps and would want Maya to connect to other sources such as Medicaid for pump access [P10].
	Maya cannot continually give the same prompts and responses. It may lead to participant disengagement from chatbots.<br><br>Some participants use chatbots to connect with live representatives while others use chatbots to avoid interacting with live representatives (aid in decreasing participant perceived burden).	

[1]P indicates participants' number.
Abbreviations: WIC: The Special Supplemental Nutrition Program for Women, Infants, and Children.
EOT: expectation of technology. EOI: expectation of information.
