# Supplementary material for: Exploring Client Perceptions on Gaining Infant Feeding Information Through the Texas Women, Infants, and Children (WIC) Chatbot
Source: Int J Environ Res Public Health. 2025 Jan 29;22(2):193. doi: 10.3390/ijerph22020193 (PMC11855084; doi:10.3390/ijerph22020193)
Supplement: Supplementary file 1 [file ijerph-22-00193-s001.zip › Supplementary Table S6.pdf]

Table S6. Comprehensive interview findings by investigated topic, Motivation.

Primary Theme	Secondary Theme	Tertiary Theme	Reflections	Quotes
**Motivation**	Necessity	Desired Nutrition Information	P4 wants nutrition information about how to prepare food for kids in a fun way to encourage eating, healthy swaps, recipes and classes.<br><br>P6 wants information to help with shopping to determine eligible items, recipes and information about introductory foods.<br><br>P9 wants Maya to help with breastfeeding issues, provide recipes, and provide information about food and/or formula allergic reactions.<br><br>P10 wants Maya to provide information about breastfeeding issues, pumping, and safety/choking hazards.	P6: "I would so love Maya to have some recipes and see ideas of what to give my baby next"<br><br>P15: "(Maya giving storage information) with breastmilk (would be helpful) because I think it's a different...it's not like powder where you can just mix it with the water when it's time."<br><br>P18: "I kind of wish (there was safety information) with breastfeeding, as far as how to clean the pumps, how to store."<br><br>P19: "(I want to be able to ask Maya) 'can my baby have nuts right now?' and she replies 'yes' or 'no'."
**Motivations**	Necessity	Perceived severity of need	P15: When prompted to list desires of chatbot features into needs, wants and don't need they stated a need for direct links to classes, wanting digital access to pamphlets and not listing anything for don't need.<br><br>P18: When prompted to list desires of chatbot into needs, wants, and don't need the participant states a need to look up benefits to avoid 'awkward situations at the cash register' and stated wanting more recipes.	P5: "I check (TexasWIC.org) every 2 to 3 days, because I like the updates with the formula and stuff as in my daughter's formula got switched. So, I like to sit there and see if (there are) any alternatives and things like that."<br><br>P8: "(WIC) gives a paper of (farmers market information) which is very beneficial. Sometimes I may lose the paper or stick it in a folder. So having a virtual copy of it would be cool. That could be a need for lots of people."<br><br>P13: "Sometimes you're like in a grocery store or something and you just want to quickly -- and if you ask someone in the shopping, they're just going to be like, I don't know. So, if she can answer that quickly for me, that would be awesome."<br><br>P13: "I think that (Maya) will help by making information readily available. Not having to wait until the WIC office

				opens. Especially if you have a question that's after hours or on the weekend."
**Motivations**	Resource	Quality of resources	P1: tried seeking information from WIC online classes regarding their child's eating style (slurping and choking). They wanted to take a WIC class because they stated trusting the classes and finding them informative. They were unable to find a live class that fits their needs and would have liked Maya to be able to answer their questions or direct them towards answers because of their stated trust in WIC.<br><br>P5: Maya is a more reliable and helpful resource because it should have a better understanding of their benefits. They state this could help them while shopping to more find eligible items specific to their package.<br><br>P14: want to access nutrition information through Maya to receive additional information and links to trustworthy external resources to bypass the perceived bias they feel during WIC appointments regarding if her child is not a picky eater or not. They state currently they seek information from google, and Facebook mom groups and have some distrust from found information but mostly trust information received through this method.	P1: "Sometimes it's going to Google other times if it's a nutrition question, I check WIC… (if I can't find it on WIC) then I will ask Google, but I haven't had that issue yet... The classes are very informative. Most of the information is already there. And I trust it over finding some random Web page."<br><br>P3: "Using WIC is more consistent (than using google) because they know what kind of ingredients I already have; so that is a big one up on helping me find recipes that I can utilize my benefits with."<br><br>P11: "I would like to have (recipes from Maya) because I trust the WIC program too -- they know the nutrition values that the kids should have and the alternatives".
**Motivations**	Resources	Source types	P1: primarily using the WIC mobile application and WIC website to seek nutrition information.<br><br>P9: using the WIC website and attending online classes to seek nutrition information.<br><br>P17: enjoy chatbots and find them convenient because they do not have to speak with someone or go into an office. They also discussed wanting Maya to be integrated into the WIC mobile application.	P8: "that's pretty cool. I didn't know about this stuff before...Maya seems very, very convenient and helpful."<br><br>P16: "I actually kind of prefer (Chatbots) sometimes…it cuts out a big step from actually talking to an actual person, when I know the artificial intelligence can get what I need done, pretty much. And it's just easier to me that way sometimes."<br><br>P18: "To search up like 'videos on baby's first foods'. That helps. I self-doubt myself a lot (about cooking), I think a

	P18: wants to speak to a person. They will first try to find a customer service number and interact with chatbots simply to redirect them to a person or live representative. However, when given a demonstration of Maya they stated that it was not a typical chatbot and were motivated to use Maya.	video would just make me more reassured I guess because I'm a more visual person."

*Indication of number of participants that provided feedback over specified topics.
Abbreviations: WIC: The Special Supplemental Program for Women, Infants, and Children.
